# Imagerie des tumeurs orbitaires chez l’enfant

**DOI:** 10.11604/pamj.2018.29.190.14671

**Published:** 2018-04-02

**Authors:** Dounia Basraoui, Fadwa Jaafari, Hicham Jalal

**Affiliations:** 1 Département de Radiologie, Hôpital Mère et Enfant, Centre d'Enseignement Mohammed VI, Faculté de Médecine de Marrakech, Université Cadi Ayad, Marrakech, Maroc

**Keywords:** Tumeurs, orbite, imagerie, enfants, Tumors, orbit, imaging, children

## Abstract

Les tumeurs orbitaires de l'enfant se caractérisent par une grande diversité histologique vue la complexité architecturale de l'orbite. Plusieurs classifications peuvent être proposées selon la localisation, le type histologique et le caractère bénin ou malin. La présentation clinique la plus fréquente est la leucocorie. Un retard diagnostique de ces tumeurs, même si elles sont bénignes, peut conditionner le pronostic et entraîner une perte de la vision et ou une déformation morphologique. L'imagerie joue un rôle important dans le diagnostic positif, la différenciation entre les processus bénins et les potentiellement malins, de faire le bilan d'extension local et loco régional et de suivre l'évolution sous traitement. L'objectif de notre travail est d'illustrer les aspects radiologiques des principales tumeurs intra-orbitaires chez l'enfant qui sont en général différentes de celles de l'adulte, à travers une étude rétrospective de 40 dossiers, colligés sur une période de 4 ans (de 2014 à 2017) au Service de Radiologie Pédiatrique du Centre Hospitalier Universitaire Mohammed VI à Marrakech au Maroc.

## Abstract (en)

Orbital tumors in children are characterised by a wide histological diversity due to the architectural complexity of the orbit. Several classifications may be proposed according to their location, histologic type and malignant or benign character. The most common clinical manifestation is the leukocoria. Diagnostic delay of these tumors, even if they are benign, may affect prognosis and lead to a loss of vision and/or a morphological deformation. Imaging plays an important role in positive diagnosis, in the differentiation between benign processes and potentially malignant processes, in the assessment of local and loco-regional staging and in follow-up monitoring under treatment. This study aimed to highlight the radiological features of primary intra-orbital tumors in children which are, in general, different from those of adults, by conducting a retrospective study of 40 medical records whose data were collected in the Division of Pediatric Radiology at the Mohammed VI University Hospital in Marrakech, Morocco over a period of 4 years (2014-2017).

## Introduction

Il existe une grande variété de tumeurs orbitaires qui diffèrent dans leurs présentations au sein de la population pédiatrique par rapport à la population adulte. Alors que la plupart de ces tumeurs sont bénignes, elles peuvent avoir un impact péjoratif sur la vision et peuvent entraîner une morbidité et une mortalité significatives. Les tumeurs orbitaires peuvent être découvertes sur l'imagerie dans de nombreuses circonstances, elles peuvent être retrouvées par hasard sur une tomodensitométrie ou une IRM cranio-faciale, réalisée pour une maladie non-ophtalmologique. En contre partie, la lésion peut être recherchée directement chez un patient présentant des signes ophtalmologiques tels qu'une exophtalmie, une acuité visuelle unilatéralement réduite, une diplopie ou une douleur orbitaire ou périorbitaire. Ces modalités d'imagerie jouent un rôle majeur dans l'évaluation diagnostique et stadiste de la lésion et orientent la décision thérapeutique. Dans cet article, nous passons en revue l'épidémiologie, les manifestations cliniques, les caractéristiques d'imagerie qui différencient les lésions orbitaires pédiatriques de celles de l'adulte, ainsi que les modalités thérapeutiques actuelles.

## Méthodes

Il s'agit d'une étude rétrospective de 40 dossiers, colligés sur une période de 4 ans (de janvier 2014 à novembre 2017) au service de radiologie pédiatrique du Centre Hospitalier Universitaire Mohammed VI à Marrakech au Maroc. Nos patients ont bénéficié d'une échographie, d'un scanner et pour certains d'entre eux d'une IRM orbitaires. Une biopsie orbitaire a été réalisée chez tous nos patients. Dans notre travail, nous allons discuter les tumeurs les plus fréquentes, avec une revue iconographique de leurs aspects les plus fréquents.

## Résultats

**Figure 1:**
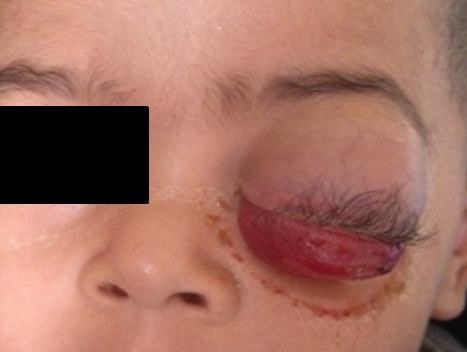
Exophtalmie avec ptosis et chémosis chez des enfants ayant un rhabdomyosarcome orbitaire

Au cours de la période d'étude, nous avons recensé dans notre centre un total de 40 cas de tumeurs orbitaires pédiatriques. L'âge moyen de nos patients était de 3 ans (de 8 mois à 14 ans), sans prédominance de sexe et sans antécédents familiaux particuliers. Le mode initial de présentation était varié, nous avons noté 61,5% d'exophtalmie (Figure 1), 28% de tuméfaction palpébrale, 14% de cécité, 10% de tuméfaction de l'angle interne de l'orbite et enfin 1 cas de chémosis et de strabisme. Nous avons recensé 17 cas de rétinoblastome soit 42,5% des tumeurs orbitaires, 13 cas derabdomyosarcome soit 32,5%, 3cas de métastases soit 7,5%, 2 cas de gliome du nerf optique soit 5%, 2cas d'hémangiome capillaire soit 5%, 2cas de lymphome soit 5% et un cas de kyste dermoide orbitaire soit 2,5%. L'exploration radiologique a été réalisée chez tous nos patients, l'échographie oculaire demandée en première intention a été réalisée chez 17 enfants, le scanner a été réalisé chez la plupart de nos patients, soit 35 cas, l'IRM a été faite chez seulement 9 patients, par faute de moyens. Les résultats de l'imagerie sont représentés dans le Tableau 1.

**Tableau 1:** Résultats des examens radiologiques

Types de tumeurs	Echographie	TDM	IRM
**Rétinoblastome**	-Tumeur solide bien limitée reliée à la rétine	-Tumeur contre la paroi avec ou sans décollement rétinien	-Iso ou hyper signal T1, hypo signal T2
	-Masse irrégulière plus échogène que le vitré	- Tumeur disséminée dans le vitré	-Prise de contraste hétérogène Extension péri-oculaire
	-Calcifications	- Rehaussement intense	
	-Décollement de rétine, hémorragie	- Calcifications+++	
**Rhabdomyosarcome**		-Masse iso dense par rapport aux muscles, assez bien limitée, prenant le contraste	-Iso ou hypo signal en séquence T1
		-Lyse des parois osseuses	-Hyper signal en séquence T2 par rapport aux muscles
		-Envahissement des cavités naso-sinusiennes et du sinus caverneux	-Rehaussement modéré à intense
**Gliome du nerf optique**		-Dilatation tumorale du nerf avec un élargissement fusiforme, régulier, iso dense à la substance grise	-Nerf optique augmenté de volume, sinueux
		- Elargissement du canal optique	- Signal identique à la substance grise en T1 et T2
		- Rehaussement modéré après injection de PDC	- Zones kystiques intra tumorales en hyper signal T2
			- Extension endocrânienne « chiasma optique»
**Hémangiome capillaire**		-Masse iso dense	-Masse avec des hypo intensités punctiformes
		- prise de contraste intense et homogène	- Signal intermédiaire en T1 entre le muscle et la graisse et en hyper signal en T2
		- Absence d’anomalies osseuse	-Prise de contraste nette
**Kyste dermoide**	-Image kystique à contours lisses, hétérogène	-Formation arrondie avec hypodensité centrale et une paroi hyperdense prenant faiblement ou pas le contraste en périphérie	-Le signal dépend du contenu du kyste, en hyper signal T1 si c’est de la graisse
	-Non vascularisé au doppler couleur	-La densité mesurée est graisseuse	-En T2, un hyper signal hétérogène intense
**Lymphome**		-Masse souvent diffuse, homogène, mal limitée, s'étendant en coulée en particulier le long de la paroi latérale de l'orbite sans atteinte osseuse	-Infiltration plus étendue touchant l'ensemble de l'espace rétrobulbaire: nerf optique, muscles et globes oculaires
			-Rehaussement modéré
			-Iso signal aux muscles en T1, hypo signal T2: non caractéristique
**Métastase**		-Formations à marges circonscrites ou mal définies, hypodenses, par rapport au muscle Calcifications	-Hypo signal sur T1, hétérogène en T2 par hémorragie ou nécrose -Prise de contraste hétérogène

## Discussion

### Tumeurs intraoculaires

**Figure 2:**
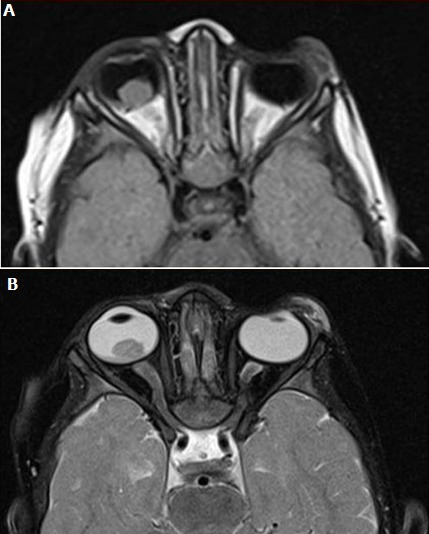
IRM cérébrale, coupes axiales, séquences pondérées en T1 (A) et T2 (B): lésion intra vitréenne postérieure droite, en regard de la macula et de la papille, en iso signal T1 et en T2: rétinoblastome de l’orbite droite

**Rétinoblastome**: Le rétinoblastome est la tumeur intraoculaire la plus commune de l'enfance, 90% des cas surviennent avant l'âge de 5 ans [1] rejoignant ainsi nos résultats. La leucocorie est le signe le plus fréquent du rétinoblastome, suivi par le strabisme [2]; dans notre étude, le signe le plus fréquent était l'exophtalmie. L'imagerie radiologique peut être utilisée pour confirmer le diagnostic et déterminer la stadification. Sur l'échographie, il peut s'agir d'une petite tumeur solide reliée à la rétine, bien limitée; ou bien une masse irrégulière qui est plus échogène que le vitrée et contient des calcifications d'ombrage, qui dans ce cas, peuvent être détectées jusqu'à 95% des cas en échographie orbitaire; on peut aussi voir un décollement de rétine ou une hémorragie [1]. Ces résultats concordaient avec les notres. L'échographie ainsi peut confirmer le diagnostic clinique du rétinoblastome [1]. Le scanner montre généralement une masse contre la paroi postérieure avec ou sans décollement rétinien, des calcifications uniques ou multiples en mottes [3] rejoignant ainsi l'aspect fréquemment retrouvé dans notre série, ou parfois une tumeur disséminée dans le vitré, parfois des lésions hyperdenses avec un rehaussement intense après injection du PDC. Il peut également établir le bilan d'extension locorégional et à distance. L'IRM évite le rayonnement ionisant du scanner et est plus sensible pour la détection de l'extension extra et péri-oculaire de la maladie, la propagation péri-neurale dans le nerf optique et l'implication de l'espace sous-arachnoïdien ainsi que l'étude du parenchyme cérébral, en particulier de la région pinéale à la recherche d'un rétinoblastome trilatéral [4], retrouvé chez un patient de notre série. Il s'agit d'une masse en iso ou hypersignal en T1, hyposignal en T2 (Figure 2), avec prise de contraste hétérogène. De ce fait, l'IRM est l'examen de choix d'évaluation et de suivi pour les cas connus de rétinoblastome avec des symptômes cliniques de propagation extra-oculaire [4].

### Tumeurs orbitaires

**Figure 3:**
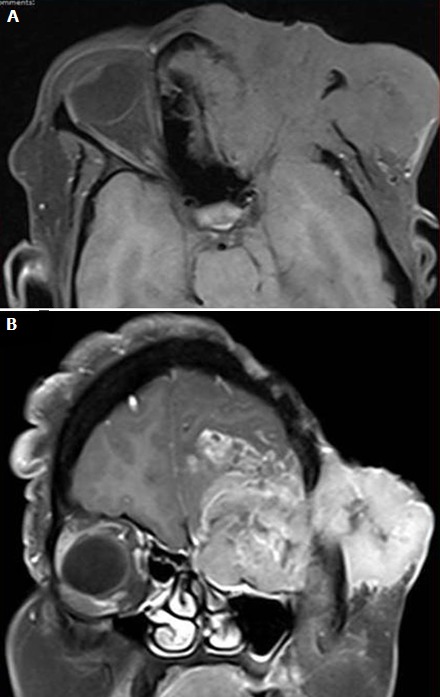
IRM cérébrale, coupes axiale pondérée en T1 (A) et coronale pondérée en T1 après injection de Gadolinium; (B) processus tumoral orbitaire gauche infiltrant, en hypo signal en T1, se rehaussant de façon hétérogène après injection du Gadolinium: Rhabdomyosarcome orbitaire

Rhabdomyosarcome: le rhabdomyosarcome est la tumeur mésenchymateuse maligne la plus fréquente chez l'enfant, avec un âge moyen de 6-8 ans [5, 6], dans notre série, l'âge moyen était de 3ans, une légère prédilection chez les garçons a été remarquée. La plupart des cas de rhabdomyosarcome sont la résultante de mutations sporadiques, par ailleurs il existe des cas qui sont associés au syndrome de Li-Fraumeni, syndrome de Beckwith-Widemann, la neurofibromatose de type 2, syndrome de Noonan et le syndrome de néoplasie endocrinienne multiple de type 2a [7]. C'est une tumeur agressive qui s'infiltre communément dans les sinus adjacents, les fissures orbitaires, le sinus caverneux et la fosse crânienne moyenne, sa propagation est hématogène, avec des métastases le plus souvent pulmonaires [4]. Le rhabdomyosarcome orbitaire se présente comme une masse indolore à croissance rapide qui conduit à l'exophtalmie [6], signe le plus fréquent retrouvé chez nos patients. En imagerie, la TDM orbitaire et l'IRM sont toutes les deux utiles pour évaluer l'étendue de la maladie et sont souvent complémentaires [4]. Le scanner montre une masse iso dense par rapport aux muscles, extra-connale, homogène et bien circonscrite, prenant le contraste de façon modérée à intense [4], aspect retrouvé dans notre série , avec des calcifications se produisant seulement si destruction osseuse, parfois l'épaississement des paupières, parfois un amincissement ou une destruction des os [4], qui peuvent être observés chez jusqu'à 40% des patients, plus rarement une nécrose, une hémorragie et une cavitation avec rehaussement en anneau. Il permet aussi d'évaluer l'envahissement des cavités naso-sinusiennes et du sinus caverneux. La TDM thoracique peut évaluer les métastases pulmonaires chez les patients atteints de rhabdomyosarcome [4]. L'IRM montre une masse en iso ou en hyposignal en séquence T1, en hyper signal en séquence T2 par rapport aux muscles avec un rehaussement modéré à intense (Figure 3). La masse peut déformer ou déplacer le globe et les muscles extra-oculaires mais envahit rarement ces structures [8]. Elle permet également d'établir le bilan d'extension périorbitaire, ainsi qu'aux sinus et aux méninges, ce qui fait changer le stade tumoral et donc la prise en charge thérapeutique et le pronostique.

**Figure 4:**
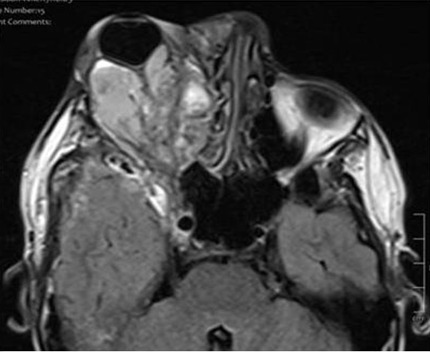
IRM cérébrale, coupes axiale pondérée en T2 Flair: processus lésionnel intra orbitaire de forme fusiforme, de contours polylobés, de signal hétérogène, siège de zones tissulaires, kystiques et hémorragiques, centré sur le nerf optique, qui est aminci et s’étend à travers le canal optique en arrière avec respect du chiasma optique: gliome du nerf optique droit

Gliome du nerf optique: le gliome du nerf optique représente 4% des tumeurs orbitaires [4], l'incidence retrouvé dans notre série était de l'ordre de 5%, il affecte les enfants entre 5 à 10 ans; environ la moitié des patients atteints de gliome de la voie optique ont une neurofibromatose (NF1). L'incidence des tumeurs chez les patients atteints de la NF1 varie de 30 à 58% et les lésions symptomatiques ne surviennent que chez 1 à 5% des patients [9]. L'implication bilatérale des voies optiques est quasi pathognomonique chez les patients atteints de la NF1 [4]. Les gliomes du nerf optique peuvent présenter une grande variété de symptômes, mais beaucoup sont asymptomatiques et sont découverts fortuitement, lors d'un dépistage par imagerie cérébrale chez les patients ayant une NF1. En revanche, l'altération de la fonction visuelle est plus souvent vue chez les patients atteints du gliome du nerf optique sporadique [10], signe difficilement mis en évidence chez nos patients vu leurs âge. La présentation clinique typique étant la diminution de l'acuité visuelle, dyschromatopsie, un défaut pupillaire afférent relatif et un champ visuel défectueux. Si les tumeurs sont grandes, elles peuvent induire un strabisme (exotropie et hypotropie) et une proptose [10]. Sur le scanner, la tumeur apparait sous forme d'une dilatation tumorale du nerf avec un élargissement fusiforme [4], signe retrouvé chez nos deux patients, régulier et iso dense à la substance grise ou parfois un élargissement du canal optique avec un rehaussement modéré après injection de PDC avec présence de zones kystiques en cas de gliome évolué [4]. L'IRM met en évidence un nerf optique augmenté de volume, sinueux à signal identique à la substance grise en T1 et en T2, parfois en hypersignal en T2, si zones kystiques intra-tumorales, les séquences injectées et les séquences T2/FLAIR peuvent aider à identifier l'étendue de l'invasion du gliome et déterminer quelles structures environnantes peuvent être impliquées (Figure 4). Elle permet également de préciser l'extension endocrânienne au chiasma optique [10]. Les gliomes de la voie optique peuvent être différenciés des méningiomes du nerf optique, car les méningiomes qui sont en hypersignal sur T2, proviennent des méninges et se rehaussent de façon intense après injection.

**Lymphome**: La maladie lymphoproliférative est rare chez les enfants [4], expliquant ainsi la faible incidence retrouvée dans notre étude qui était de 0,5%, représentant moins de 5-10% des lésions orbitaires. L'hyperplasie lymphoïde représente 10 à 40% et le lymphome non hodgkinien représente 60 à 90% des lésions [4], l'atteinte est uni ou bilatérale. Ces lésions sont le plus souvent observées au niveau du quadrant supéro-temporal avec une prédilection pour la glande lacrymale; cependant, ils peuvent présenter une atteinte musculaire extra oculaire diffuse pouvant imiter l'ophtalmopathie thyroïdienne [11]. Sur le plan clinique, les présentations sont variables en fonction du site de l'atteinte, mais le plus souvent il s'agit d'une exophtalmie progressive non douloureuse non ou peu inflammatoire [12], signe retrouvé chez nos patients; une vision floue, une baisse de l'acuité visuelle et des corps flottants sont les symptômes subjectifs initiaux communs. Sur le scanner on retrouve une masse souvent homogène, mal limitée, s'étendant en coulée en particulier le long de la paroi latérale de l'orbite sans atteinte osseuse [12], même aspect retrouvé chez nos patients, parfois l'élargissement des glandes lacrymales ou des muscles extra oculaires. Différencier la maladie lymphoproliférative d'une pseudotumeur inflammatoire est difficile sur le scanner [4] car les deux maladies peuvent provoquer l'élargissement des glandes lacrymales ou des muscles extra oculaires. L'IRM met en évidence une infiltration plus étendue touchant l'ensemble de l'espace rétrobulbaire, soit le nerf optique, les muscles et le globes oculaire, en iso signal en T1par rapport aux muscles et en hypo signal en T2 (non caractéristique) [4]; cet aspect a été retrouvé chez un seul patient chez qui on avait réalisé l'IRM. L'hyperplasie lymphoïde et le lymphome ont également des caractéristiques d'imagerie qui se chevauchent. L'hyperplasie peut sembler plus circonscrite et le lymphome peut apparaître plus infiltrant [11].

**Kyste dermoïde**: C'est la 1ère cause de masse orbitaire chez l'enfant (50%) [13]; dans notre série, un seul cas de kyste dermoide a été recensé. Il touche les enfants entre 1 à 4 ans. Développé à partir d'un tissu ectodermique résiduel contenant des éléments dermiques (graisse, glandes sébacées). Il n´est pas commun dans les cavités orbitaires et intracrâniennes; cependant, aucun organe du corps n'est immunisé. Il doit être pris en compte dans le diagnostic différentiel des lésions kystiques orbitaires telles que le tératome, le choristome (épidermoïde et dermolipome), le kyste colobomateux et l'œil kystiquecongénital. Sa présentation clinique dépend de l'emplacement, de la taille, du taux de croissance, de l'extension intracrânienne et de la corrélation avec les structures adjacentes [13]. Les signes cliniques sont variés et vont d'une masse avec gonflement des paupières [4] retrouvés également chez notre patient, ptosis, au déplacement du globe, proptose, à la protrusion oculaire, limitation de la motilité oculaire et syndrome de compression du nerf optique [4]. La clinique et l'échographie suffisent la plupart du temps pour poser le diagnostic en objectivant une image kystique à contours lisses, d'échogénicité variable sans aucune vascularisation interne démontrable [14], rejoignant la même description retrouvée chez notre patient. Sur le scanner, il s'agit souvent d'une formation arrondie bien circonscrite uniloculaire, siège d'une hypodensité centrale avec une paroi hyperdense prenant faiblement ou pas le contraste en périphérie, sa densité est graisseuse, pas d'ostéolyse mais un défect osseux est fréquent, souvent situé en regard d'une suture [14]. À l'IRM, le signal dépend du contenu du kyste; en hypo signal T1 si c'est de la kératine et en hyper signal si c'est graisseux, un niveau liquidien est possible. En T2, on note le plus souvent une formation en hyper signal hétérogène. La taille est en général supérieure ou égale à 1cm [4].

### Les lésions vasculaires

**Hémangiome capillaire**: Tumeur vasculaire bénigne due à une prolifération de l'endothélium vasculaire, elle est très fréquente [6], sa prévalence au niveau de notre série était de l'ordre de 5%. Diagnostiquée dans les premières semaines voir les premiers mois de vie [6], avec une prédominance féminine. Classée en pré-septal, intra orbitaire (impliquant l'orbite post septal), et composé ou mixte, impliquant le pré et post septal. L'évolution se fait le plus souvent vers une régression spontanée [6]. L'attitude thérapeutique est l'abstention dans les formes simples, la chirurgie retrouve sa place dans les formes très volumineuses avec un risque d'obturer l'axe visuel [4]. L'imagerie à pour rôle d'évaluer la profondeur de la lésion et ses rapports avec les structures adjacentes pour toute lésion présentant des caractéristiques atypiques à l'examen ou à la présentation physique, ou un syndrome sous-jacent associé [4]. Le scanner montre une masse iso dense avec prise de contraste intense et homogène sans anomalies osseuses [4], l'aspect retrouvé chez nos patient était une masse homogène, infiltrante, adhérente aux tissus environnants avec extension postérieure en « doigt de gants» et infiltration des paupières, se rehaussant de façon intense après injection de produit de contraste. À l'IRM, Les hémangiomes typiques sont des masses solides bien circonscrites avec des vides d'écoulement artériel, en signal intermédiaire sur T1 et en hyper signal sur T2, avec une prise de contraste nette. Au cours de la phase d'involution, les vides et l'écoulement diminuent [4].

**Les métastases**: Rares chez l'enfant, avec une clinique aspécifique. Les atteintes topographiques préférentielles sont les muscles oculomoteurs, la gaine du nerf optique, la choroïde et la grande aile du sphénoïde. Le neuroblastome est l'origine fréquente des métastases orbitaires chez l'enfant avant l'âge de 2ans; 8 à 20% des cas de métastases orbitaires [4]. Elles peuvent être asymptomatiques ou découvertes lors du bilan d'extension ou révélatrices. L'atteinte est bilatérale dans 40% des cas. Le sarcome d'Ewing vient en deuxième rang chez le grand enfant ou l'adolescent [4], retrouvé chez un seul enfant de notre série contre deux ayant des neuroblastome métastatiques. En dernier le néphroblastome donne très rarement des métastases orbitaires [15]. Le neuroblastome métastatique donne sur le scanner des formations à marges circonscrites ou mal définies, hypodenses, par rapport au muscle, parfois on note la présence de calcifications. La TDM permet d'évaluer l'envahissement des structures adjacentes [16]. À l'IRM, elles sont en hypo signal sur T1, hétérogène en T2 par hémorragie ou nécrose, avec prise de contraste hétérogène. Elle permet également d'établir le bilan d'extension de la tumeur par voie intracrânienne et au niveau des tissus mous adjacents [16].

## Conclusion

Un large assortiment de néoplasmes intraoculaires et de masses orbitaires affecte l'orbite pédiatrique et peut présenter diverses manifestations cliniques. Une approche systématique et multidisciplinaire est nécessaire pour faciliter un diagnostic précis. Le rétinoblastome et le rhabdomyosarcome constituent les tumeurs les plus fréquentes dans notre contexte. L'imagerie notamment le scanner dans notre contexte, permet d'orienter le diagnostic étiologique et de guider la prise en charge thérapeutique. L'IRM est d'un grand apport dans le bilan d'extension et dans le suivi post thérapeutique.

### Etat des connaissances actuelle sur le sujet

- Il existe une grande variété de tumeurs orbitaires, même si la plupart sont bénignes, mais elles peuvent avoir un impact péjoratif sur la vision et peuvent entraîner une morbidité et une mortalité significatives;
- L'imagerie joue un rôle majeur dans l'évaluation diagnostique et stadiste de la lésion et orientent la décision thérapeutique.

### Contribution de notre étude à la connaissance

- Les tumeurs orbitaires diffèrent dans leurs présentations cliniques et radiologiques au sein de la population pédiatrique par rapport à la population adulte, le but de notre travail était de mettre le point sur ces particularités.

## Conflits d’intérêts

Les auteurs ne déclarent aucun conflit d'intérêts.
